# Next-generation sequencing to dissect hereditary nephrotic syndrome in mice identifies a hypomorphic mutation in *Lamb2* and models Pierson’s syndrome

**DOI:** 10.1002/path.4308

**Published:** 2014-02-06

**Authors:** Katherine R Bull, Thomas Mason, Andrew J Rimmer, Tanya L Crockford, Karlee L Silver, Tiphaine Bouriez-Jones, Tertius A Hough, Shirine Chaudhry, Ian SD Roberts, Christopher C Goodnow, Richard J Cornall

**Affiliations:** 1 Nuffield Department of Medicine and Wellcome Trust Centre for Human Genetics, Oxford UniversityUK; 2 MRC Human Immunology Unit, Weatherall Institute for Molecular Medicine, Oxford UniversityUK; 3 Department of Immunology, John Curtin School of Medical Research, Australian National UniversityCanberra, Australia; 4 MRC Harwell, Harwell Science and Innovation CampusOxfordshire, UK; 5 Australian Phenomics Facility, Australian National UniversityCanberra, Australia; 6 Oxford University Hospitals NHS Trust, John Radcliffe Hospital, Headington, OxfordUK

**Keywords:** proteinuria, nephrotic syndrome, congenital nephrosis, kidney glomerulus, glomerular basement membrane, next-generation sequencing, mutagenesis, animal model

## Abstract

The study of mutations causing the steroid-resistant nephrotic syndrome in children has greatly advanced our understanding of the kidney filtration barrier. In particular, these genetic variants have illuminated the roles of the podocyte, glomerular basement membrane and endothelial cell in glomerular filtration. However, in a significant number of familial and early onset cases, an underlying mutation cannot be identified, indicating that there are likely to be multiple unknown genes with roles in glomerular permeability. We now show how the combination of *N*-ethyl-*N*-nitrosourea mutagenesis and next-generation sequencing could be used to identify the range of mutations affecting these pathways. Using this approach, we isolated a novel mouse strain with a viable nephrotic phenotype and used whole-genome sequencing to isolate a causative hypomorphic mutation in *Lamb2*. This discovery generated a model for one part of the spectrum of human Pierson’s syndrome and provides a powerful proof of principle for accelerating gene discovery and improving our understanding of inherited forms of renal disease. Copyright © 2013 Pathological Society of Great Britain and Ireland. Published by John Wiley & Sons, Ltd

## Introduction

Twenty percent of paediatric patients with the nephrotic syndrome fail to achieve remission with steroid treatment 1, and these patients have a high incidence of progression to end-stage kidney disease 2. In contrast to the more common form of the disease, the steroid-resistant nephrotic syndrome (SRNS) is predominantly monogenic, involving at least 24 known genes 3, including *NPHS1* 4, *NPHS2* 5, *WT1* 6, *LAMB2* 7, *CD2AP* 8, *PLCE1* 9, *ACTN4* 10, *TRPC6* 11 and *INF2* 12. These targets have highlighted the key role of the podocyte in maintaining the integrity of the glomerular filtration barrier 13 and the importance of the endothelial cell and the extracellular glomerular basement membrane (GBM), which is composed of collagen IV, laminin, heparan sulphate proteoglycans and nidogen-1, in maintaining selective permeability 14,15. The mutations causing SRNS have revealed diverse and sometimes unexpected functions, ranging from ion channels to the organization of the actin cytoskeleton or mitochondrial function. In 20–40% of familial cases of childhood-onset SRNS 16–18 and at least 30% of all cases within the first year of life 19,20, the underlying gene is not known. Combined with the diversity of genes and pathways already identified, this suggests there are many unknown genes contributing to the filtration barrier, with potential as therapeutic targets 21.

Whilst future studies using next-generation sequencing will undoubtedly identify more of the genes involved in familial cases of SRNS, such families are rare, and it is therefore possible that a simultaneous and complementary approach using animals could accelerate the discovery of new genes and provide models for human disease. In particular, forward screens using the chemical mutagen *N*-ethyl-*N*-nitrosourea (ENU) are a powerful method to identify genes without *a priori* assumptions as to pathways or function, by inducing random point mutations that mimic human variation. The aim of this study was to establish an efficient method to identify genetic variants causing proteinuria in a mouse model, using ENU mutagenesis. By harnessing whole-genome sequencing (WGS), we were able to rapidly isolate the causative mutation in an ENU pedigree with the nephrotic syndrome.

## Materials and methods

### ENU and phenotyping

*Nephertiti* mice were generated at the Australian Phenomics Facility, Australian National University, Canberra. C57BL/6 J (B6) founder mice were treated with three doses of 90–100 mg/kg *N*-ethyl-*N*-nitrosourea (Sigma), as previously described 22. Proteinuria was detected by urine dipstick (Multistix, Bayer Health Care) in third-generation (G3) offspring from such a founder.

### Collection of blood samples

Mice aged 17–25 weeks were terminally anaesthetized and blood samples were collected, by cardiac puncture, into lithium heparin paediatric tubes and centrifuged to separate out the plasma.

### Routine clinical chemistry of plasma samples

Clinical chemistry was performed on a Beckman Coulter AU400 semi-automated clinical chemistry analyser by the Mary Lyon Centre’s clinical pathology service laboratory at MRC Harwell. All assays were carried out using the manufacturer’s instructions, parameter settings and reagents. Samples were analysed for total protein, albumin, total cholesterol, triglycerides urea and creatinine. Electrolytes (sodium, potassium and chloride), total calcium, inorganic phosphate, alanine aminotransferase, aspartate aminotransferase, alkaline phosphatase, HDL cholesterol, LDL cholesterol and glucose were also measured.

### Histology

Tissue sections were fixed in 10% neutral buffered formalin, embedded in paraffin, cut to 3 µm sections and stained with either haematoxylin and eosin (H&E), periodic acid–Schiff (PAS) or methenamine silver, according to published methods. For transmission electron microscopy, tissue was fixed with gluteraldehyde, embedded with resin and sectioned at 70 nm. The Oxford Centre for Histopathology Research performed the electron microscopy and silver staining. IgG immunofluorescence was performed on formalin-fixed tissues, following pronase antigen retrieval 23 using fluorescein isothiocyanate (FITC)-conjugated IgG (Caltag) 24. 4′,6-Diamidino-2-phenylindole (DAPI) was used for nuclear counterstaining. Immunofluorescence images were captured using a Zeiss 510 metahead confocal microscope.

### DNA extraction

DNA was extracted from tail tissue using a DNAeasy kit (Qiagen), and quantified using a Qubit fluorometer (Invitrogen).

### Conventional mapping

B6 mice were out-crossed to the CBA/J strain for mapping and bred to bring the causative mutation to homozygosity, using dipstick urine testing to track the phenotype. Linkage mapping was performed using simple sequence-length polymorphisms (SSLP) and single nucleotide polymorphisms (SNPs) (Figure 2A, B). Maximal LOD scores 25 using a range of recombination fractions < 0.5 were calculated at each of 26 polymorphic loci, given the observed alleles in a mean of 27 affected and unaffected mice from the *nephertiti* pedigree.

### Whole-genome sequencing and mapping

Whole-genome sequencing was performed on one lane of a HiSeq 2000 (Illumina) to 5.8× mean coverage, using paired-end 100 bp reads. Reads were mapped with Stampy 26 against the MGSCv37 mouse reference genome; 92.7% of the genome was covered by at least one read. Variants were called with Platypus (Rimmer A, Mathieson I, Lunter G, McVean G, (2012) Platypus: An Integrated Variant Caller http://www.well.ox.ac.uk/platypus) and filtered against an in-house union file of variants seen in other ENU mouse pedigrees, and against dbSNP v 128. The variants were also filtered for coverage depth, allelic or strand bias, homopolymers, repetitive sequence and quality of local variation. In-house Python scripts were used for all filtering steps.

An in-house algorithm based on a hidden Markov model 27 was used to infer the most likely ancestral haplotypes inherited by the sequenced *nephertiti* mouse, based on the observed frequency of variants and knowledge of the ENU mutation rate 28; for details, see Supplementary material. A bam format file containing the read data for this experiment will be made freely available in the European Nucleotide Archive data repository.

### Validation of the *Lamb2* mutation with Sanger sequencing

Whole genomic DNA from the sequenced mouse was amplified using oligonucleotide primers (forward CTATGCTGGTGGAGCGTTCT, reverse TGAGTAGCGGGACTCACACA) and Biotaq polymerase (Bioline Reagents Ltd). Amplification of the PCR product was carried out using BigDye reagents (Applied Biosystems) and sequenced on an ABI Prism 3100 machine.

### Statistical analyses

LOD score calculations were as described above, all other statistical analyses were performed using the GraphPad Prism package. WT-to-mutant *p* values for clinical chemistry were calculated using unpaired two-tailed *t*-tests.

### Ethics

All animal experiments were approved by local and national ethical review, including the Australian National University Animal Ethics and Experimentation Committee and the Oxford University Local Ethical Review Committee and UK Home Office (License No. PPL 30/2455).

## Results

**Figure 1:**
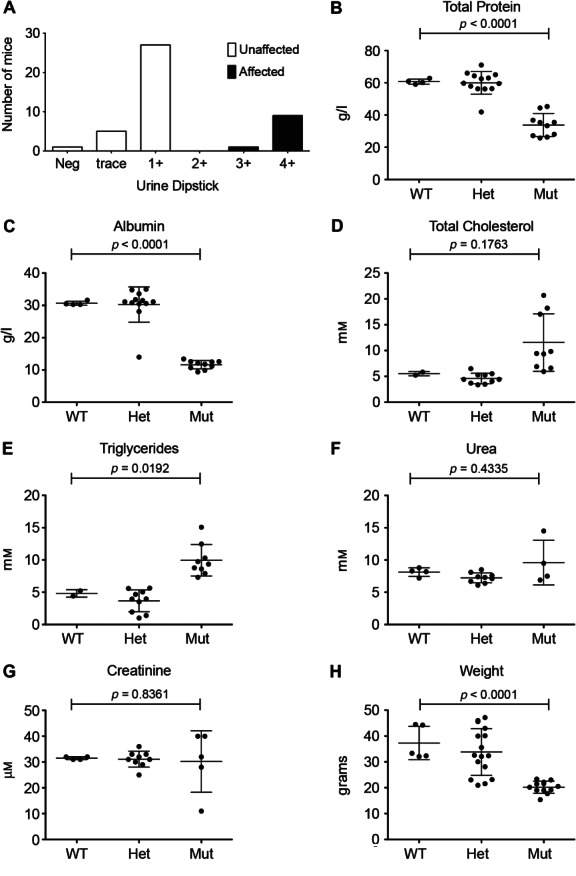
*Nephertiti*, an ENU mutant strain with proteinuria. (A) Urine protein in affected *nephertiti* mice and unaffected littermates, where 3 + = 3–10 mg/ml and 4+ > 10 mg/ml. Comparison of plasma total protein (B), albumin (C), total cholesterol (D), triglycerides (E), creatinine (F) and weight (G) in age-matched, homozygous, heterozygous and WT sibling controls, between 17 and 25 weeks. *p* values are based on unpaired two-tailed *t*-tests between WT and homozygous mutants. Error bars indicate mean and standard deviation

**Figure 2:**
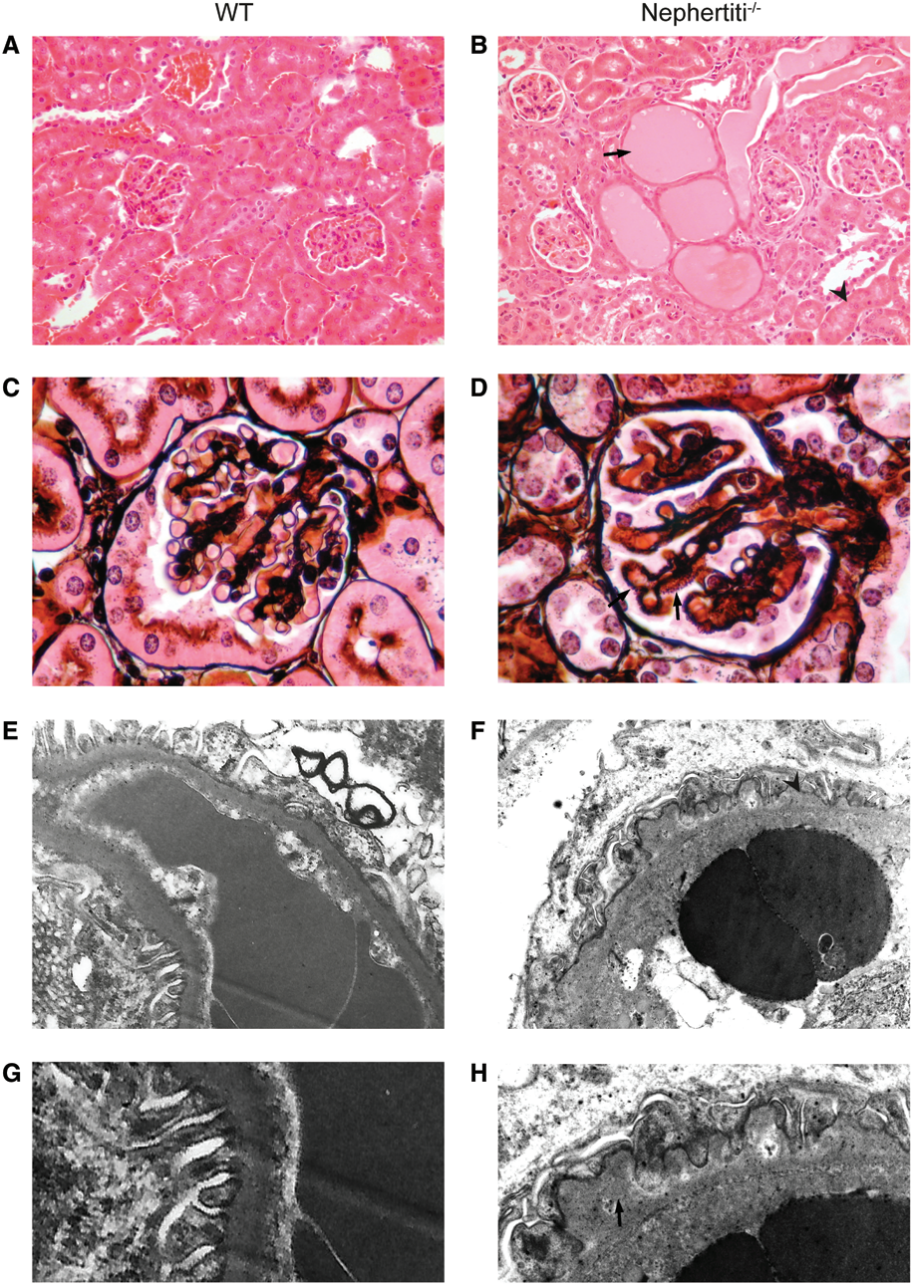
Renal light microscopy and electron microscopy in WT (A, C, E, G) and *nephertiti* (B, D, F, H) mice. (A, B) After H&E staining, at objective magnification ×20, *nephertiti* mice show dilated renal tubules containing protein casts (arrow) and protein resorption droplets in tubular epithelium (arrowhead). (C, D) After methenamine silver staining, at objective magnification ×60, *nephertiti* mice show prominent membrane spikes (arrow). (E, F) By electron microscopy, at magnification ×18 500, *nephertiti* mice show irregularly thickened glomerular basement membranes (arrowhead). (G, H) Higher-power images of basement membrane in (E, F), with subepithelial spikes and podocyte foot process effacement in *nephertiti* (arrow). All histology is shown in homozygous affected *nephertiti* mice and WT unaffected siblings. Histology is representative of samples from three affected and three WT for each stain

In the course of a programme screening third-generation offspring of ENU-treated mice for immune phenotypes 29, we detected several mice from a single pedigree with heavy proteinuria on dipstick testing, which was detectable at weaning (3 weeks of age) and inherited in an autosomal recessive manner (Figure 1A). The *nephertiti* mouse strain exhibited a nephrotic phenotype. Mice examined between 17 and 25 weeks had hypoproteinaemia and hypoalbuminaemia, raised cholesterol and trigylcerides and low body weight in comparison to wild-type (WT) B6 mice. Urea and creatinine were not significantly raised in comparison to WT (Figure 1B–G). Light microscopy of the kidneys of affected animals, but not WT littermates, demonstrated protein casts within tubules and protein resorption droplets within tubular epithelium (Figure 2A, B). Methenamine silver staining revealed a coarsely thickened GBM with widespread basement membrane spikes (Figure 2C, D). Electron microscopy (EM) showed an irregular appearance to the subepithelial GBM with areas of thickening and spikes. The subendothelial GBM surface remained smooth. There was moderate effacement of the podocyte foot processes (Figure 2E–H). Immunofluorescence confirmed the absence of IgG antibody accumulation (see supplementary material, Figure S1).

**Figure 3:**
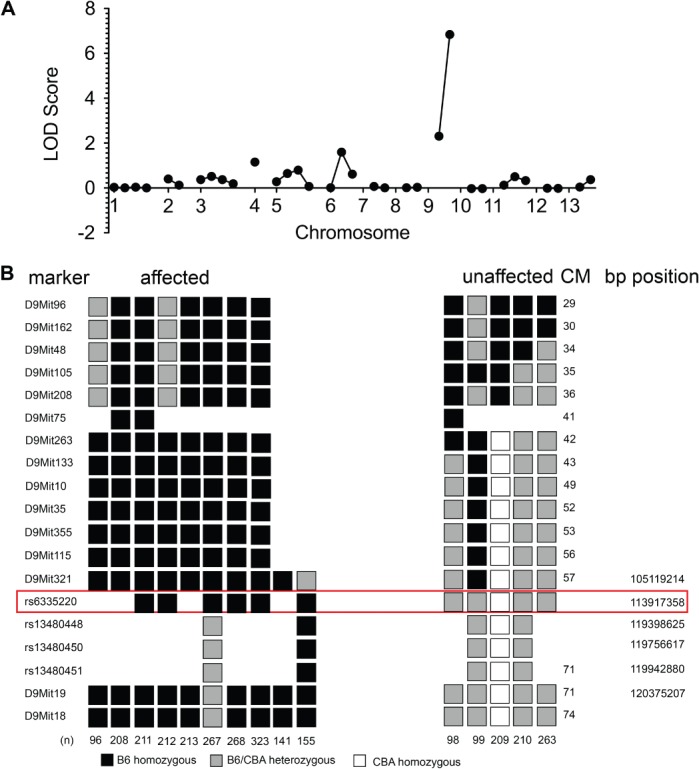
Mapping the *nephertiti* mutation to chromosome 9. (A) Coarse mapping using SSLP. (B) Fine mapping using SSLP and SNPs. The only SNP with fully consistent linkage to the homozygous phenotype is shown boxed; the limits of the linkage region are defined by the adjacent SNPs

Due to the random nature of ENU mutagenesis, out-crossing to another inbred laboratory strain and linkage mapping using strain-specific polymorphisms has conventionally been required to identify the causative mutation. Consequently, *nephertiti* was out-crossed to the CBA/J strain and bred to homozygosity, tracking the phenotype by urinalysis. Coarse linkage mapping identified simple sequence-length polymorphisms (SSLPs) with LOD scores of 6.84 and 2.3 on chromosome 9 (Figure 3A). Fine mapping narrowed the candidate region to 14.3 Mb on chromosome 9 (Figure 3B), containing 311 RefSeq or 559 Ensembl gene annotations.

We then carried out low-coverage WGS in order to identify the causative mutation. DNA from one affected mouse was sequenced to mean 5.8× coverage. Because the mouse had been out-crossed, there were > 3.8 × 10^6^ variants called; however, excluding low-quality calls and known variation reduced this number to 298 876 (see Materials and methods). Within the 14.3 Mb linkage region there were a total of 1680 variants, of which eight were in coding regions or splice sites. Filtering for those affecting protein sense (missense, nonsense or splice variant) and homozygous in the affected mouse reduced this to one candidate, a G–A transition at position 108 383 650 on chromosome 9, predicted to result in a C185Y amino acid substitution in exon 5 of the laminin-*β*2 protein.

Mutations in human *LAMB2* cause Pierson’s syndrome, which typically presents with severe nephrotic syndrome alongside ocular abnormalities and neuromuscular hypotonia 7. Since *Lamb2* knock-out mice exhibit nephrotic range proteinuria 30, both the clinical and animal data suggest that this mutation is causative in *nephertiti*.

**Figure 4:**
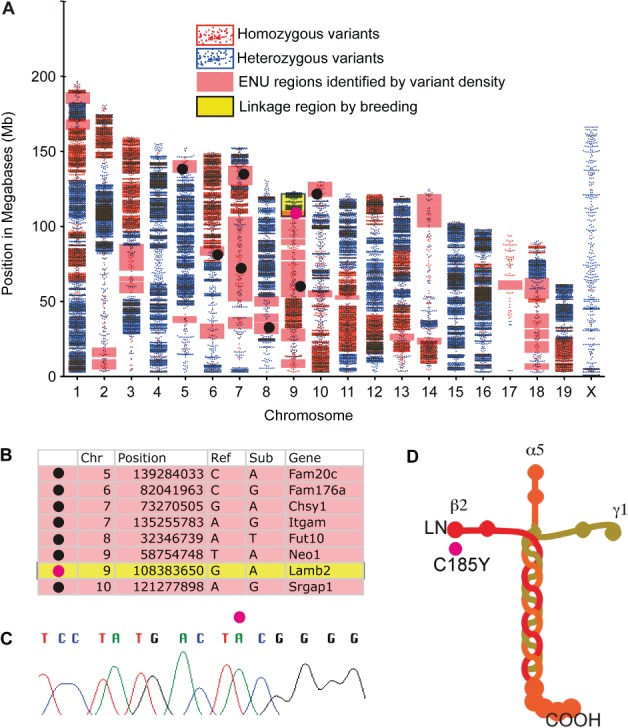
A hypomorphic laminin *β*2 mutation is the cause of proteinuria in *nephertiti*. (A) Scatter plots of homozygous (red) and heterozygous (blue) filtered WGS variants for each chromosome. High-density regions correspond to the CBA genomic regions; regions inheriting both alleles from the ENU-treated founder have a lower density of homozygous variation than CBA regions but higher than background variation from the reference in WT genomic intervals. The region identified by conventional linkage mapping is shaded yellow. Regions identified as homozygous ENU in this mouse by variant density using the hmm algorithm are shaded pink. Black circles indicate candidate coding variants affecting protein sense, the *Lamb2* variant is shown as a pink circle. (B) Candidate variants identified by WGS without reference to the conventional linkage region include the causative mutation. (C) Sanger sequencing confirms the *Lamb2* mutation. (D) The mutation induces a C-to-Y substitution in the globular N-terminal domain (LN) of laminin *β*2, on the *β*2 short arm of the laminin 521 heterotrimer

Although this result demonstrated that WGS combined with linkage data could isolate the causative mutation, we wanted to examine the efficacy of a WGS approach without utilizing conventional linkage information. To do this, we developed an algorithm to identify all genomic intervals inherited from the ENU-treated founder, based on the density of variants (see Supplementary material). The algorithm identified regions comprising 263 Mb as having two alleles inherited from the ENU-treated founder (Figure 4A). These regions contained 347 of the filtered variants. Selecting for homozygous variants affecting protein sense reduced the number of candidates to eight (Figure 4B), including the *Lamb2* mutation. Sanger sequencing confirmed the mutation (Figure 4C), which is highly conserved and predicted deleterious by Polyphen-2 31.

## Discussion

Laminin forms the basic scaffold protein of basement membranes, as cruciform heterotrimers composed of *α*, *β* and *γ* subunits. The principal laminin of adult GBM is composed of *α*5, *β*2 and *γ*1 subunits 32 and is also expressed at the neuromuscular synapse 33. The mutation in *nephertiti* lies in the globular N-terminal domain on the short arm of laminin *β*2, which is important for trimer polymerization 34 (Figure 4D).

*Lamb2^−/−^* mice have severe neuromuscular disease, which contributes to their perinatal lethality; but when transgenic laminin *β*2 is expressed in skeletal muscle (MCK-B2), the mice develop proteinuria and die of kidney failure at 1 month 35. In contrast, the *nephertiti* mice survive beyond 6 months. All mice homozygous for the *nephertiti* allele had 3+ or 4+ protein on dipstick testing (Figure 1A), consistent with 3–10 mg/ml or > 10 mg/ml urinary protein, respectively 36, and comparable with the 10 mg/ml reported in *Lamb2*^−/−^-null mice 37. Therefore, the prolonged survival in *nephertiti* mice appears to be due to preserved renal function despite nephrosis (Figure 1F, G), and different from the effects of the null mutation 38. This would be consistent with a hypomorphic mutation, with mutant laminin *β*2 expressed in the GBM, and suggests that the defect in *nepheriti* mice may be due to abnormal function of the mutant protein.

The *nephertiti* phenotype is consistent with the spectrum of human Pierson’s syndrome. Patients with truncating null mutations develop mesangial sclerosis and end-stage renal failure within the first few years of life, often combined with complex ocular abnormalities and severe psychomotor retardation 39. However, several groups have identified patients with missense mutations in *LAMB2*, which are associated with slower renal progression and in some cases an absence of extrarenal manifestations 40–43. The latter would be consistent with the phenotype in *nephertiti*, where survival, breeding, locomotor function and behaviour appear to be normal. The human missense mutations are clustered at the globular N-terminal domain 44, and some have been shown to result in low levels of laminin *β*2 in the GBM 42. In contrast, damaging premature stop codons are distributed across the gene, and deletion of even the last 39 amino acids results in complete loss of protein expression 7, presumably because the C-terminus is required for trimer assembly 45.

The mechanism by which missense mutations in the N-terminal domain induce the nephrotic syndrome is not fully understood. Polymerization through binding of these domains is mediated via C-terminal interactions with dystroglycan and integrin receptors *in vitro*, driving reciprocal cytoskeletal assembly and actin reorganization 46 and suggesting that correct polymerization of laminin could involve podocyte signalling. The filtration barrier failure observed with podocyte-specific knockout of the integrin-linked kinase *ILK* also points to a role for the interaction between laminin in the GBM and podocyte integrin 47. The prominence of membrane spikes in *nephertiti* closely resembles mice lacking the tetraspanin *CD151*, in which lack of integrin *α*3*β*1–CD151 interactions may also result in podocyte detachment and compensatory production of excess GBM components 48. The fact that over-expression of laminin *β*1 can partially rescue the *Lamb2^−/−^* phenotype 49, albeit an incomplete rescue, despite adequate laminin trimer formation, indicates that laminin *β*2 has unique properties, perhaps due to its higher binding avidity to integrin *α*3*β*1 compared with laminin *β*1 50.

Proteinuria precedes foot process effacement in *Lamb2^−/−^* mice, suggesting that the defect is at least partly GBM-intrinsic^38^. Consistent with this, we found relative preservation of podocyte structure in *nephertiti*, despite GBM thickening and the nephrotic syndrome. Changes in one component of the GBM can affect others, for example in Alport’s syndrome, where mutations in collagen IV result in ectopic expression of laminin 51; however, *Lamb2^−/−^* mice have normal collagen IV chain expression 30. A transgenic N-terminal mutant laminin-*β*2 only partially rescues the *Lamb2^−/−^* phenotype, with low levels of *β*2 at the GBM attributed to failure to secrete the protein 52, and moderate transgene expression resulting in a moth-eaten, irregularly thickened GBM, similar to *nephertiti*. The transgenic C321R mutation results in misfolding in the endoplasmic reticulum and death due to renal failure by 3 months 53. The preserved function and prolonged survival in *nephertiti* may be due to the more physiological expression of the mouse protein; alternatively, the C185Y mutation, which unlike C321R lies N-terminal to the disulphide-bonded cysteines of the EGF-like repeats, may have lesser consequences for protein folding.

Whereas *nephertiti* was generated on a B6 background, the targeted knockout *Lamb2^−/−^* was originally from 129 s1/SvJ ES cells and could therefore carry 129 specific modifiers in linkage to the *Lamb2* gene on a B6 background. Although formal proof would require analysis of a *Lamb2^−/−^* on a pure B6 background, we think it unlikely that linked genes in the congenic region have important modifier effects: first, because it is possible to effect a partial rescue of the renal-specific *Lamb2^−/−^* mice, originally from 129 ES cell origin, with a B6CBAF2/J mutant transgene 52; and second, because we did not observe any phenotypic consequences following outcrossing to the CBA/J strain for mapping in *nephertiti*.

Laminin is thought to be produced by both podocytes and endothelial cells in the glomerulus 54; however, glomerular hybrid experiments suggest that cellular origin influences laminin localization within the GBM 55. Access to a mutant strain expressing a hypomorphic form of laminin *β*2 under its physiological promoter will make it possible to isolate such cell-specific effects.

ENU mutagenesis coupled to high-throughout sequencing offers two advantages for the discovery of new genes involved in the development or maintenance of the glomerular filtration barrier, which are illustrated by our study. First, point mutations induce viable phenotypes that mimic human disease, so *nephertiti* models the milder spectrum of Pierson’s syndrome and confirms the importance of the N-terminal domain in laminin *β*2 function. Second, it is now possible, using WGS, to move rapidly from phenotypic screens for proteinuria, urinary protein:creatinine ratio, renal clearance, histology or imaging to the identification of causative mutations. Future screens based on these principles will identify novel genes, variants and pathways involved in heritable forms of human renal disease and provide tools for investigating the underlying mechanisms that cause pathology.

## Supplementary Material on the Internet

The following supplementary material may be found in the online version of this article

Details of the algorithm to identify genomic intervals inherited from the ENU founder

Figure S1 Expression of IgG in the kidney
