# Supplementary material for: Next-generation sequencing to dissect hereditary nephrotic syndrome in mice identifies a hypomorphic mutation in Lamb2 and models Pierson’s syndrome
Source: J Pathol. 2014 Feb 6;233(1):18–26. doi: 10.1002/path.4308 (PMC4241031; doi:10.1002/path.4308)
Supplement: Supplementary file 1 — Details of the algorithm to identify genomic intervals inherited from the ENU founder [file path0233-0018-sd1.doc]

**<Supplementary material>**

+A: **Simultaneous identification of the genomic intervals and candidate mutations inherited from the ENU-treated founder**

In order to shortlist candidate causative mutations in *nephertiti* without recourse to conventional linkage mapping, it is necessary to distinguish the ENU-induced homozygous mutations from the large amount of homozygous variation from the reference genome in genomic regions inherited from the CBA/J ancestor. This is achieved by identifying regions of the sequenced *nephertiti* mouse genome inherited from the B6 ENU-treated founder by density of variation. Plotting the variants by genomic location across each chromosome demonstrates the densely clustered variation in CBA/J genomic intervals, and less dense variation in intervals inherited from the ENU-treated B6 founder (Figure 4A). In order to more precisely define these intervals, we developed an algorithm based on a hidden Markov model (HMM).

A HMM is a general framework for making inferences about unobserved states from observed data [1]. Here the observed data are the sequence of variant calls and their density. The underlying states correspond to the six possible genotypes, ENU/ENU (homozygous for ENU-treated founder), CBA/CBA (homozygous for the outcross strain), WT/WT (homozygous for the untreated C57BL/6J reference strain), ENU/WT, ENU/CBA and CBA/WT.

The model is able to discriminate between genomic intervals inherited from the different founders, due to large differences in variant density. We assume an ENU frequency of 1.5 SNPs/Mb, based on our own findings over a large number of variants in other sequenced ENU mice [2]. We empirically derive a WT background variant frequency of 0.2 SNPs/Mb and CBA to reference strain variant frequency of 200 SNPs/Mb.

In order to infer the most likely sequence of ancestral haplotypes across each chromosome, we use the Viterbi algorithm and incorporate the average mouse recombination frequency of 0.56 cM/Mb per meiosis [3], the distance between variants and the number of recombinant alleles.

All in house scripts are coded in Python (www.python.org).

+A: **Mapping and variant calling settings and versions**

Mapping: Stampy version 1.0.12 was run with bwaoptions.

Variant calling: Platypus v 0.1.9 was used (www.well.ox.ac.uk/platypus).

Settings were as follows:

'minReads': 2, 'refFile': 'ref_files/Mouse37', 'maxHaplotypes': 256, 'filterVarsByCoverage': 1, 'maxSize': 250, 'parseNCBI': 0, 'ploidy': 2, 'numPasses': 1, 'useIndelErrorModel': 0, 'nCPU': 1, 'minFlank': 3, 'bufferSize': 1000000, 'useEMLikelihoods': 0, 'logFileName': 'log.txt', 'regions': None, 'maxVariants': 8, 'maxReads': 5000000, 'badReadsWindow': 11, 'genIndels': 1, 'maxVarDist': 25, 'abThreshold': 0.050000000000000003, 'minMapQual': 20, 'rlen': 100, 'minGoodQualBases': 20, 'maxEMIterations': 100, 'maxBadQualBases': 20, 'nInd': 1, 'getVariantsFromBAMs': 1, 'genSNPs': 1, 'minPosterior': 5, 'minVarFreq': 0.20000000000000001, 'verbosity': 2, 'sourceFile': None, 'filteredReadsFrac': 0.69999999999999996, 'badReadsThreshold': 15, 'sbThreshold': 0.050000000000000003, 'mergeClusteredVariants': 1, 'minBaseQual': 20}

+A: **Supplementary references**

1. Rabiner LR. A tutorial on hidden Markov models and selected applications in speech recognition. *Proc IEEE* 1989; **77**(2): 257–286.

2. Bull KR, Rimmer AJ, Siggs OM, *et al.* Unlocking the bottleneck in forward genetics using whole-genome sequencing and identity by descent to isolate causative mutations. *PLoS Genet* 2013; **9**(1): e1003219.

3. Jensen-Seaman MI, Furey TS, Payseur BA, *et al.* Comparative recombination rates in the rat, mouse, and human genomes. *Genome Res* 2004; **14**(4): 528–538.
