# Supplementary figures and images for: Next-generation sequencing to dissect hereditary nephrotic syndrome in mice identifies a hypomorphic mutation in Lamb2 and models Pierson’s syndrome
Source: J Pathol. 2014 Feb 6;233(1):18–26. doi: 10.1002/path.4308 (PMC4241031; doi:10.1002/path.4308)

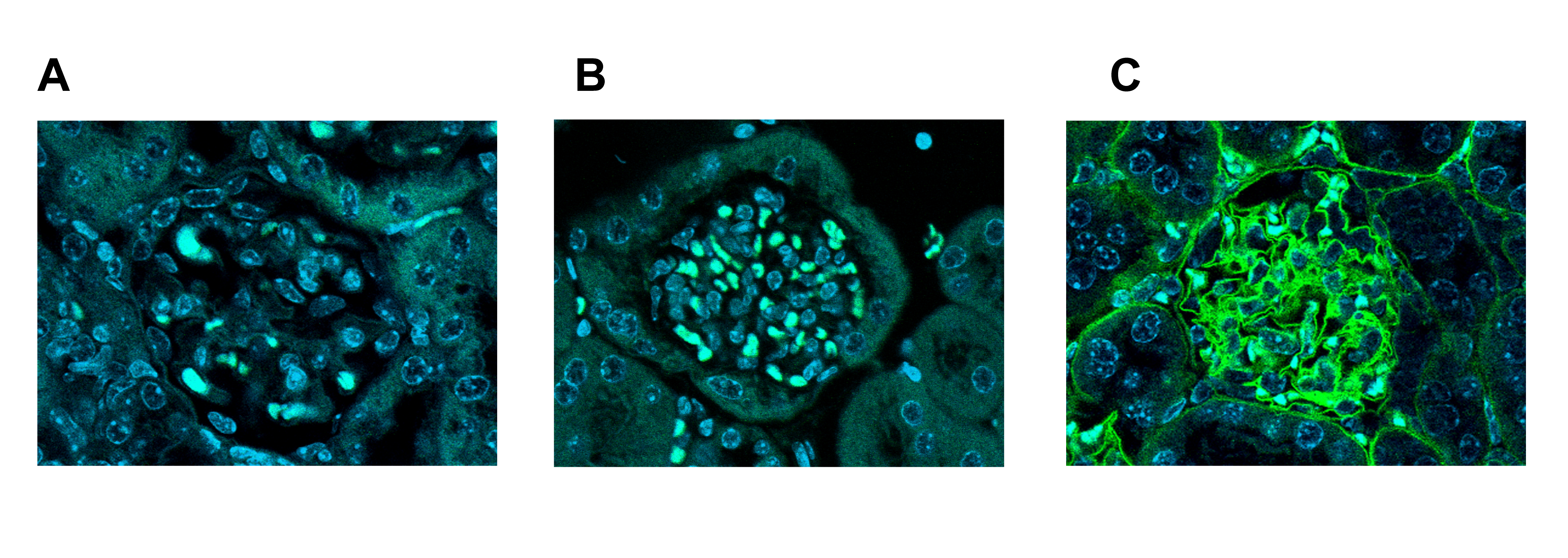

Supplement: Figure S1 — Expression of IgG in the kidney [file path0233-0018-sd2.tif]
